# Novel Psychoactive Substances and Behavioral Addictions

**DOI:** 10.1155/2014/534523

**Published:** 2014-12-28

**Authors:** Giovanni Martinotti, Ornella Corazza, Sophia Achab, Zsolt Demetrovics

**Affiliations:** ^1^Department of Neuroscience and Imaging, “G. d'Annunzio” University of Chieti-Pescara, Italy; ^2^School of Life and Medical Sciences, University of Hertfordshire, Hatfield, UK; ^3^Department of Mental Health and Psychiatry, University Hospitals of Geneva, Switzerland; ^4^Institute of Psychology, Eötvös Loránd University, Budapest, Hungary

The present special issue addresses two key areas, which at first sight may not appear to be particularly related to each other. Their novelty, however, connects them tightly. The appearance of these two issues has, on one hand, required and promoted a renewal of the perspective in addiction science and, on the other hand, could facilitate the rethinking of practical, therapeutic, and preventive interventions. Thus, both areas share a main common aspect: they raise new issues that need to be addressed in the field of addictions and, as such, force us to reconsider former approaches.

One of the focuses is the emergence of novel psychoactive substances, especially in relation to the fast moving and the potentially unlimited nature of their online market [1]. A “novel psychoactive substance” has been legally defined by the European Union as a new narcotic or psychotropic drug, either in pure form or in a preparation, that is not scheduled under the Single Convention on Narcotic Drugs of 1961 or the Convention on Psychotropic Substances of 1971, but which may pose a public health threat comparable to substances listed in the above-mentioned conventions (Council of the European Union decision 2005/387/JHA) (UNODC, 2014).

From the psychedelic movements of the 1960s until the beginning of the 21st century, there was hardly any change regarding the most used drugs. In addition to cannabis, heroin, and cocaine, primarily LSD and, to a lesser extent, magic mushrooms among hallucinogenic drugs and amphetamine basically covered the significant range of substances of abuse. Although the presence of ecstasy from the end of the 1980s had brought some moderate changes, it can be stated that researchers and prevention and treatment professionals could work in a relatively stable environment throughout decades. Users basically used the same drugs and sought help for similar problems, and therefore studies could systematically grow and support the preventive and clinical efforts.

The novel psychoactive substances, however, that began to spread after the millennium [2–4] have fundamentally reorganized this apparently stable situation. Since then, new substances have constantly been appearing on the market. It is not rare that there is no time to even identify a new drug before another substance takes its place on the market and they might disappear a few days, weeks, or months later, while newer ones appear. This extremely rapidly changing situation concerning drug use and drug market set new challenges for professionals. We have to describe the use of drugs on which we have very limited knowledge. Not only is the chemical description of these substances often unavailable, but often we do not even know their street names. The effects of drugs should be explored while their name and nature are unknown to both the dealer and the user. We have to estimate the risks associated with substances without being familiar even with their most basic characteristics. We have to provide information to potential users of these drugs and we hardly know anything about them or treat unknown side effects or overdoses.

It is no surprise that in this rapidly changing environment initiatives have been launched [5] that apply new data collection methods and aim to gather information as fast as possible and share this knowledge within target groups via new effective tools [2, 3]. The opportunities provided by the developments in the field of info communication technologies, the Internet, and smartphones could be of great value for this aspect. All these factors, in fact, contribute to the rethinking and renewal of data collection and data analysis methods, the reallocation of resources, and the reconsideration of prevention tools and efforts, as well as the implementation of other types of interventions. The present special issue, on one hand, intends to make a contribution for facing these challenges.

On the other hand, during the past few years, “new addictions” have emerged in addition to the new drugs. These addictions are actually not a new problem of course, but the formerly described disorders appear in a new interpretative framework. In the past decades, but especially in the past few years, the opinion that the range of addictive disorders should not be reduced to psychoactive substances-related dependencies has been strengthened. In line with this, scientific literature on addictions has begun to deal with a growing number of phenomena, originally not classified as addictive disorders, but with important psychological and social consequences [6–8].

Among behavioral addictions gambling disorder represents the main area, giving the large diffusion of gambling. With the increase in availability of gambling products across the world and the increased exposure to gambling advertisements both on TV and on mobile platforms, we are currently faced with a larger number of people across the globe who are using gambling as a recreational pursuit.

Issues of current significance in the gambling world are the targeting of children and adolescents by the gambling industry, the targeting of older people by larger casinos offering company and distraction but focusing on a vulnerable group, and the targeting of female and indigenous populations, who are being reached by specific advertising campaigns across most of the world.

Besides the new interpretation of gambling disorder, formerly classified as an impulse control disorder, new interpretations of numerous other disorders from the addiction perspective have also been raised. Among these are other impulse control disorders, eating disorders, specific sexual disorders, and, among other problems officially not classified as disorders, the Internet addiction, online gaming, exercise addiction, and many others. It is becoming more and more common to see the expressions of behavioral addiction or nonsubstance use addiction [9, 10]. The formal expansion of the range of addictive disorders to the direction of addictions not related to chemical substances has eventually been brought by the publishing of DSM-5 [11]. In this edition of the manual, the former section on “*Substance-Related Disorders*” has been replaced with “*Substance-Related and Addictive Disorders.*” This chapter indeed includes a subchapter titled “*Non-Substance-Related Disorders.”* Although addiction experts could classify several disorders belonging here, at the moment the only official member of this group is gambling disorder. This modification, however, is of great symbolic significance: it clearly represents the shift towards a new perspective in which the presence of a psychoactive substance is not a prerequisite to addictive disorders.

*Giovanni  Martinotti* *Sophia  Achab* *Ornella  Corazza* *Zsolt  Demetrovics*
